# Trends in molecular epidemiology of drug-resistant tuberculosis in Republic of Karelia, Russian Federation

**DOI:** 10.1186/s12866-015-0613-3

**Published:** 2015-12-18

**Authors:** Igor Mokrousov, Anna Vyazovaya, Natalia Solovieva, Tatiana Sunchalina, Yuri Markelov, Ekaterina Chernyaeva, Natalia Melnikova, Marine Dogonadze, Daria Starkova, Neliya Vasilieva, Alena Gerasimova, Yulia Kononenko, Viacheslav Zhuravlev, Olga Narvskaya

**Affiliations:** Laboratory of Molecular Microbiology, St. Petersburg Pasteur Institute, 14 Mira street, St. Petersburg, 197101 Russia; Laboratory of Etiological Diagnostics, Research Institute of Phthisiopulmonology, St. Petersburg, Russia; Republican Tuberculosis Dispensary, Petrozavodsk, Republic of Karelia Russia; Petrozavodsk State University, Petrozavodsk, Republic of Karelia Russia; Theodosius Dobzhansky Center for Genome Bioinformatics, St. Petersburg State University, St. Petersburg, Russia

**Keywords:** *Mycobacterium tuberculosis*, Spoligotyping, Beijing genotype, Russia, Karelia, Finland, Phylogeography

## Abstract

**Background:**

Russian Republic of Karelia is located at the Russian-Finnish border. It contains most of the historical Karelia land inhabited with autochthonous Karels and more recently migrated Russians. Although tuberculosis (TB) incidence in Karelia is decreasing, it remains high (45.8/100 000 in 2014) with the rate of multi-drug resistance (MDR) among newly diagnosed TB patients reaching 46.5 %. The study aimed to genetically characterize *Mycobacterium tuberculosis* isolates obtained at different time points from TB patients from Karelia to gain insight into the phylogeographic specificity of the circulating genotypes and to assess trends in evolution of drug resistant subpopulations.

**Methods:**

The sample included 150 *M. tuberculosis* isolates: 78 isolated in 2013–2014 (“new” collection) and 72 isolated in 2006 (“old” collection). Drug susceptibility testing was done by the method of absolute concentrations. Spoligotyping was used to test genotype-specific markers of a Latin-American-Mediterranean (LAM) family and its sublineages as well as a Beijing B0/W148-cluster.

**Results:**

The largest spoligotypes were SIT1 (Beijing family, *n* = 42) and SIT40 (T family, *n* = 5). Beijing family was the largest (*n* = 43) followed by T (*n* = 11), Ural (*n* = 10) and LAM (*n* = 8). Successful Russian clone, Beijing В0/W148, was identified in 15 (34.9 %) of 43 Beijing isolates; all В0/W148 isolates were drug-resistant. Seven of 8 LAM isolates belonged to the RD115/LAM-RUS branch, 1 - to the LAM RD174/RD-Rio sublineage. MDR was found in Beijing (32/43), Ural (3/10), and LAM (3/8). In contrast, all T isolates were pansusceptible. Comparison of drug resistant subgroups of the new and old collections showed an increasing prevalence of the B0/W148 clonal cluster, from 18.0 % (mainly polyresistant) in 2006 to 32.6 % in 2014 (mainly MDR and pre-XDR). The West–east increasing gradient is observed for the Ural genotype that may be defined a ‘Russian’ strain. In contrast, the spoligotype SIT40 of the T family appears to be a historical Karelian strain.

**Conclusions:**

Circulation of the MDR *M. tuberculosis* isolates of the Beijing genotype and its B0/W148 cluster continues to critically influence the current situation with the MDR-TB control in northwestern Russia including the Republic of Karelia. Revealed phylogeographic patterns of some genotypes reflect a complex demographic history of Karelia within the course of the 20^th^ century.

## Background

Tuberculosis (TB) in Russia remains a major national health problem. However, one of the markers of an unequal social and economic development of different regions within the country is the disparity with regard to the burden of TB. The Republic of Karelia is located at the Russian-Finnish (de facto European Union) border (800 km) and contains most of the historical Karelia land. It occupies 180 520 sq. km and has a population of 632 533. Currently Karelia has high TB incidence of 45.8/100,000 in 2014 (although decreased from 62.3 in 2009) and very high level of mortality compared to the Russian North-West as a whole (24 % higher in 2008) [1, 2]. The situation is seriously aggravated by increase in prevalence of multidrug-resistant (MDR) *Mycobacterium tuberculosis* strains: from 15.4 % in 2006 to 46.5 % in 2014 in newly-diagnosed TB group (two-fold higher than nationally) and from 64.1 % to 73.3 % among previously-treated patients (T. Sunchalina, pers. comm.). Since the collapse of the USSR, the population mobility via Finnish-Karelian border has been intensified [3, 4] which may have some impact on the epidemiology of TB in Finland.

The study aimed to perform a molecular analysis of *M. tuberculosis* isolates obtained at different time points (2006 vs 2013–2014) from Karelian TB patients in order to gain insight into the phylogeographic specificity of the circulating genotypes and assess their role in spread of drug resistant TB.

## Methods

### Bacterial strains

In total, 150 *M. tuberculosis* isolates were analyzed. Firstly, a “new” collection included 78 strains collected prospectively from June 2013 to January 2014 in different regions across Karelia. Of them, 69 were isolated from newly diagnosed TB patients and constituted all isolated from this patient category within the survey period. Secondly, 71 strains (mainly drug-resistant) isolated from newly diagnosed TB patients in 2006 made up a retrospective convenience sample (“old” collection).

*M. tuberculosis* susceptibility to the first-line (isoniazid, rifampicin, pyrazinamide, ethambutol, streptomycin) and second-line (ofloxacin, cycloserine, amikacin, kanamycin, capreomycin, ethionamide) anti-TB drugs was done using a method of absolute concentrations [5] and/or BACTEC MGIT 960 system according to the manufacturer’s recommendations. The bacteriology laboratory at the Tuberculosis Dispensary in Petrozavodsk is externally quality assured by the Federal System for External Quality Assessment in Laboratory Medicine of Russian Federation. The isolates were defined as multidrug-resistant (MDR), extremely drug resistant (XDR) and pre-XDR according to the WHO definitions.

### Ethical approval

The study was approved by Ethical Board of St. Petersburg Research Institute of Phthisiopulmonology. All patients gave informed written consent stating that they agree to the anonymous use of their clinical and epidemiological information and biological samples (DNA of *M. tuberculosis* strain) for the study.

### Genotyping

*M. tuberculosis* DNA was extracted using a recommended method [6]. DNA was subjected to spoligotyping as described [7]; spoligotyping profiles were compared to the SITVIT_WEB database at http://www.pasteur-guadeloupe.fr:8081/SITVIT_ONLINE/query.

Latin-American Mediterranean (LAM) family was detected by testing specific *Rv0129c* SNP; LAM isolates were tested for RD115, RD174, RD-Rio, and LAM-RUS markers using assays described previously [8–10]. Beijing B0/W148 cluster was identified by testing its specific *Rv2664-Rv2665*::IS*6110* insertion [11]. MIRU26 VNTR locus was specifically tested on ambiguous Ural/Haarlem spoligoprofiles; 1 copy in MIRU26 is considered a marker of the Ural family [12]. Otherwise, Ural family was identified by specific spoligotype signature [13].

A chi-square test was used to detect any significant difference between the two groups. Yates corrected χ2 and *P*-values were calculated with 95 % confidence interval at http://www.medcalc.org/calc/odds_ratio.php online resource.

## Results

### Molecular characteristics of the new collection, 2013–2014

A total of 78 *M. tuberculosis* isolates obtained from 2013 to 2014 represented patients from different districts across Russian Karelia. All patients were permanent residents in Karelia, of Caucasian (“white”) ethnicity. Of those 5 were homeless and the others had a registered place of residency in urban or rural settings. Most of the patients were newly diagnosed (*n* = 69), 9 were retreatment cases. Fifty-six were male, 22 were female (which makes a usual 2.5:1 gender ratio of TB). Mean age was 43.4 years (range: 22–87 years, SD ± 13.9), in male group - 43.9 years (range: 22–87 years, SD ± 14.3), in female group - 42.3 years (range: 28–82 years, SD ± 13.2).

To outline the population structure at *M. tuberculosis* family/subfamily level, the isolates were subjected to the combination of several molecular typing methods. Based on spoligotyping, 78 isolates were subdivided into 8 types shared by 2 to 42 isolates and 16 singletons (Table 1). Some types were not found in the published version of the SITVIT database. Interestingly, the 2nd largest type (after Beijing SIT1, 42 isolates) was SIT40 (T family) that included 5 isolates.

**Table 1:**
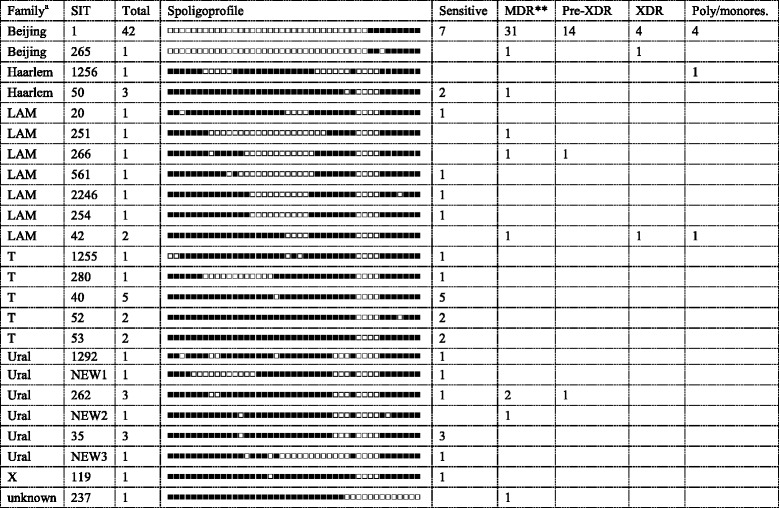
Spoligotypes and drug resistance profiles of *M. tuberculosis* isolates, 2013-2014

^a^Family assigned by SITVIT_WEB and corrected by use of other markers. SIT20 is RD-Rio, other LAM SIT are LAM-RUS

^b^MDR includes also pre-XDR and XDR

Based on spoligotyping and other molecular markers, 78 isolates were assigned to the following families: Beijing (*n* = 43), “ill-defined” T family (*n* = 11), Ural (*n* = 10), LAM (*n* = 8), Haarlem (*n* = 4), X (*n* = 1), and unknown family (*n* = 1) (Table 2). Of 43 Beijing isolates, 14 belonged to the B0/W148-cluster previously termed as Russian successful clone [11]. Interestingly, Ural sample was small enough but quite diverse even at the level of spoligotyping: 10 isolates presented 6 different profiles. LAM family included 7 isolates of RD115 sublineage (all belonged to its LAM-RUS branch) and 1 isolate of RD174/RD-Rio sublineage (a SIT20 isolate).

**Table 2:** Distribution of drug resistance across *M. tuberculosis* families, 2013–2014

Family, cluster^a^	All	Sensitive	MDR^b^	Pre-XDR	XDR	Poly/monores.
Beijing	43	7	32	14	5	4
B0/W148-cluster	15		14	6	2	1
Other Beijing	28	7	18	8	3	3
T	11	11				
Ural	10	7	3	1		
LAM	8	4	3	1	1	1
LAM-RUS	7	3	3	1	1	1
Haarlem	4	2	1			1

^a^Single isolates were of X (sensitive) and unknown family (MDR) and are not shown

^b^MDR includes also pre-XDR and XDR

Thirty-two isolates were susceptible to all tested drugs, 6 isolates were monoresistant (of them 5 were STR-resistant) and 41 isolates were resistant to two and more drugs. The resistant isolates were subdivided into MDR, pre-XDR and XDR groups and stratified by genetic family (Table 1, Table 2). The Beijing family was associated with the high rate of drug resistance: 32 of 43 Beijing family strains were MDR (this includes also pre-XDR and XDR isolates). Multidrug resistance was also found in the LAM (3 of 8 isolates) and the Ural (3 of 10) genotypes. In contrast, all 11 isolates of the T family were sensitive to all tested drugs. One should note that the Beijing B0/W148 variant included only drug resistant isolates (8 of 15 were pre-XDR or XDR) while the other Beijing variants (*n* = 28) included 11 pre-XDR/XDR and 7 sensitive isolates.

Within the new collection the MDR rate among all genotypes (Table 2) shows an association with MDR for Beijing family (32/43 vs 7/35 other genotypes pooled together; *P* < 0.0001). Data on resistance to the second-line drugs also show an association between the Beijing family and pre-XDR and XDR profile: 19/43 Beijing family vs 3/35 other genotypes’ isolates, *P* = 0.0016).

### Molecular characteristics of the retrospective collection, 2006

The available retrospective collection of 71 strains isolated in 2006 presented a convenience sample that included mostly drug resistant isolates (60 isolates were resistant and 11 were susceptible). Within this study, it was used to assess changes in the epidemiology of drug resistant subpopulation of *M. tuberculosis* in Karelia when compared with new collection described above.

Two patients were homeless while the rest had a registered place of residence. Fifty-two patients were male, 19 were female. Mean age was 43.0 years (range: 21–72 years, SD ± 12.5); in the male group - 42.7 years (range: 21–72 years, SD ± 11.7), in the female group - 43.9 years (range: 24–72 years, SD ± 14.8).

The isolates were subjected to the same typing methodology as described above for the new collection. The distribution of genotypes within the entire collection and across different drug resistance categories is shown in Tables 3 and 4. The Beijing family was represented by a single spoligotype SIT1 (67.6 % of the entire collection). It was followed by more diverse LAM (12 isolates, 7 profiles) and T (5 isolates, 5 profiles) families. Most profiles received SIT numbers through comparison with SITVIT_WEB while 4 profiles were not found in this database.

**Table 3:**
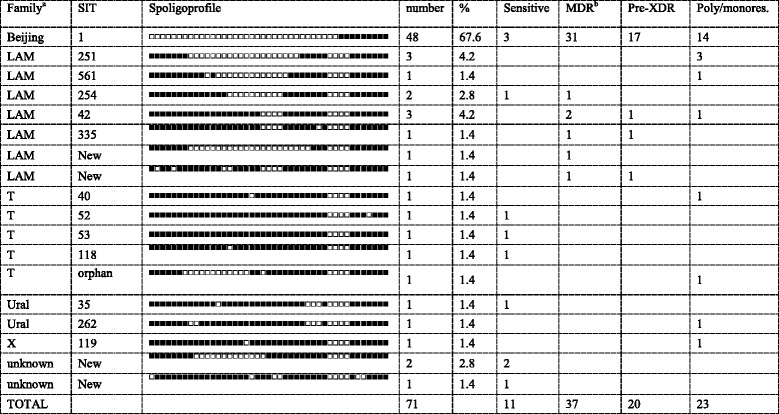
Spoligotypes and drug resistance profiles of *M. tuberculosis* isolates, 2006

^a^Family was assigned by SITVIT_WEB and corrected by use of other markers

^b^MDR includes also pre-XDR

**Table 4:** Drug resistant and susceptible *M. tuberculosis* isolates, 2006

Family, cluster	All	Sensitive	MDR^a^	Pre-XDR	Poly/monores.
Beijing, *n* = 48	48	3	31	17	14
B0/W148-cluster, *n* = 11	11	ᅟ	4	3	7
Other Beijing, *n* = 37	37	3	27	14	7
T, *n* = 5	5	3			2
Ural , *n* = 2	2	1			1
LAM, *n* = 12	12	1	6	3	5
X, *n* = 1	1				1
Unknown, *n* = 3	3	3			

^a^MDR includes also pre-XDR

Almost one-fourth of the Beijing isolates belonged to the B0/W148 cluster. All LAM isolates belonged to the RD115 sublineage and its LAM-RUS branch.

Multi-drug resistance was identified only in the isolates that belonged to the Beijing and LAM families (Table 4). Majority of the Beijing (31/48) and half of the LAM family strains were MDR. In case of the LAM family, MDR was not associated with any particular spoligotype. Although XDR was not identified in any of those isolates, 17 of 48 Beijing and 3 of 12 LAM were pre-XDR.

### Comparison of drug resistant subpopulations from two time periods

A retrospective collection of 72 strains isolated in 2006 was mainly drug resistant and we looked at the two time points separately for drug resistant (60 vs 46 isolates) and susceptible (11 vs. 32 isolates) subgroups (Tables 2 and 4).

Quantitative and qualitative trends observed in drug resistant subgroups, from 2006 to 2014, show some increase in the prevalence of the Beijing genotype from 73.8 % (45/61) to 78.3 % (36/46). However a closer look reveals that this increase was exclusively due to the increasing rate of the B0/W148 clonal cluster, from 18.0 % (mainly polyresistant) to 32.6 % (mainly MDR and pre-XDR), although borderline non-significantly (*P* = 0.08). The B0/W148 strains were absent in fully susceptible groups from both collections. The prevalence of other, non-B0 Beijing strains was similar within the resistant groups: 39.3 % (24/61) and 39.1 % (18/46) in 2006 and 2014, respectively.

A comparison of drug susceptible subgroups from the 2006 and 2014 collections showed that T-family isolates did not acquire additional resistance; its prevalence was similar among susceptible isolates (3/11 in 2006 and 11/32 in 2014). However this observation should be interpreted with caution due to the lack of clarity in definition of the T family as a whole.

## Discussion

Epidemiology of tuberculosis in a given area is greatly influenced by social and economic situation, health policy as a whole and implementation of National program against TB in particular. No less it is defined by the local population structure of the circulating *M. tuberculosis* strains: an increasing prevalence of highly virulent clones may severely jeopardize efforts of health authorities. Two collections of strains collected with 8-year gap, were used to perform a phylogeographic analysis in the context of neighboring countries and Russian provinces and in the light of human migration and changing demographics, and to assess molecular epidemiology and dynamic changes of the drug resistant *M. tuberculosis* subpopulations in Karelia.

### Phylogeography

Past migration of human populations in this area in Northern Europe and historically more recent trans-border exchange shaped both human and human pathogens’ local population structures. In modern Karelia the proportion of Karels (autochthonous people close to Finns) decreased from 37.4 % in 1926 to 7.4 % in 2010. This occurred through massive influx from the neighboring Russian regions between 1920s and 1940 and from more distant and diverse areas across the USSR in 1946–1954 [14]. Furthermore, the borders and population of the Soviet Republic of Karelia have undergone changes since 1920s through (i) incorporation of the ethnically Russian districts, and (ii) annexation of parts of Finnish Karelia by the Soviet Union in 1940/1944 [15, 16].

**Fig. 1:**
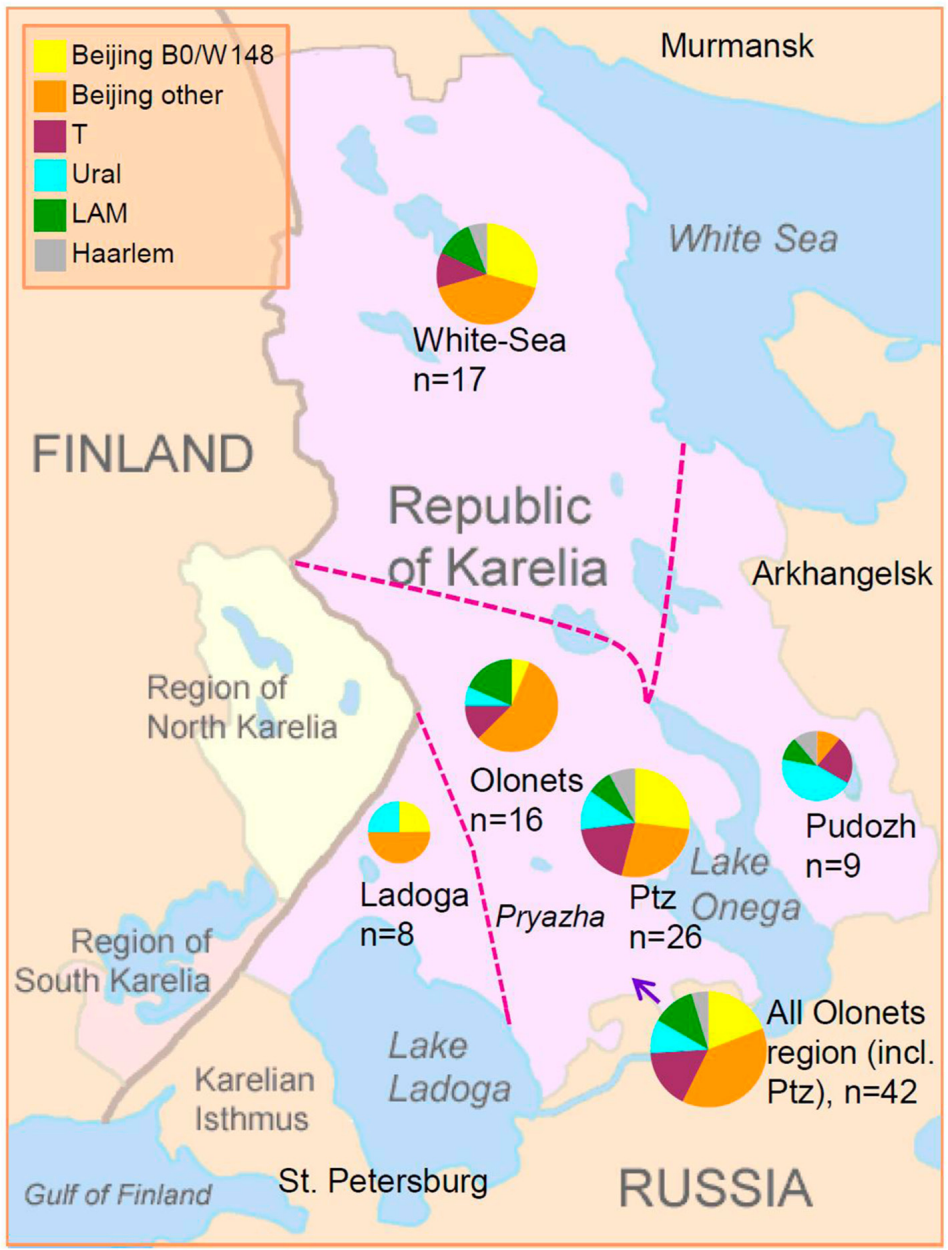
Distribution of *M. tuberculosis* genotypes (collection 2013–2014) in different regions of Karelia Circle size is roughly proportional to the sample size. Ptz = Petrozavodsk. Free map: http://commons.wikimedia.org/wiki/File:Karelia_today.png

**Fig. 2:**
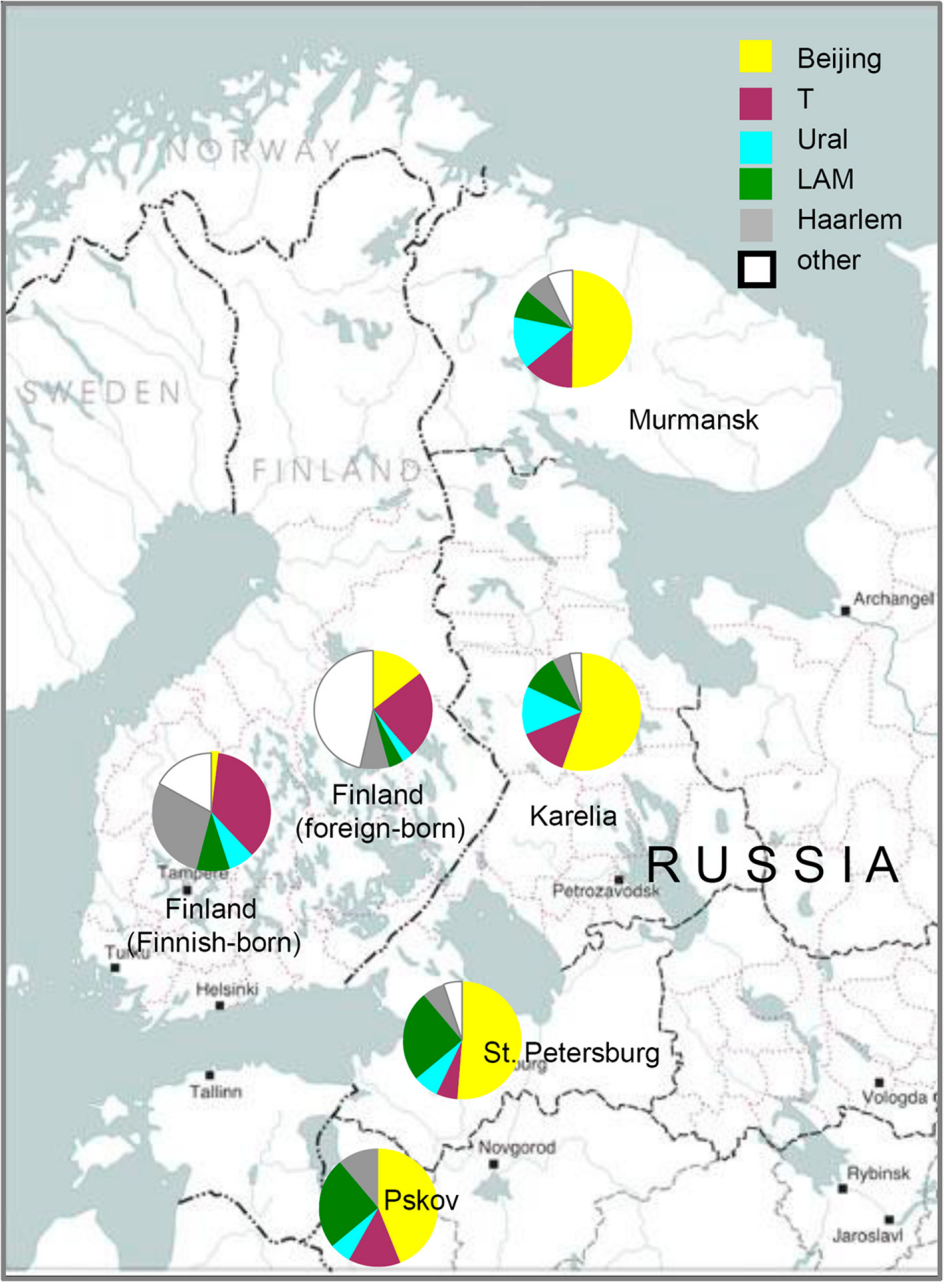
Distribution of *M. tuberculosis* genotypes in Karelia (collection 2013–2014) in its neighboring regions in Russia, and in Finland. Circle size is not proportional to the sample size. The same color coding is used as in Fig 1, but Beijing genotype is shown by a single yellow color, without separate shading of the Beijing B0/W148 variant. Free map: http://s8366.chomikuj.pl/ChomikImage.aspx?e=6c-SLnsUnfRfdfr7pJzD_Ks6q2jz1mmsSXERXBmYqw0C3H5OBm9Si_yGn7ckP54SV8PouVOQiyMi4FM5truhxxxGI_tRJhzFlrimtfVsbns&pv=2

Distribution of genetic families across historic regions within Karelia is shown on the map (Fig. 1), it was also compared to the neighboring areas (Fig. 2; Tables 5 and 6) [11, 17–27] although different study design was a limitation.

**Table 5:**
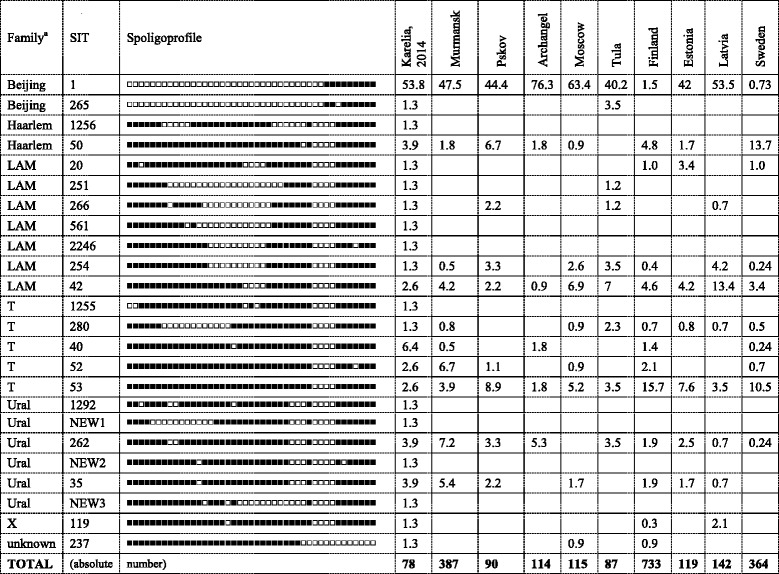
Spoligotypes identified in this study (2013–2014 sample) and in neighboring areas, %

^a^Family assigned by SITVIT_WEB and corrected by use of other markers. SIT20 is RD-Rio, other LAM SIT are LAM-RUS

Previously published collections represent: Finland (Finnish-born [17]), Estonia (old sample - 2000 and earlier [23]), Latvia (old sample and mainly drug resistant [27]), Sweden (patients born before 1945 [21]), Pskov, Russia [18], Murmansk, Russia [24], Tula, Russia (prison setting [22]), Moscow, Russia (mainly MDR [19]), Archangel, Russia (prison setting [26])

**Table 6:** Distribution of genotype families identified in *M. tuberculosis* in Karelia (2013–2014 sample) in its neighbors (% of total population)

Family, cluster	Karelia^a^, this study, year 2014, *n* = 78	St. Petersburg	Pskov, *n* = 90	Murmansk, *N* = 387	Finland (Finnish-born), *n* = 733	Finland (foreign-born), *n* = 264
Beijing	55.1	51	44.4	50	1.5	15.2
B0/W148-cluster	19.2	13	8	5.5 (MDR sample)		
Other Beijing	35.9	38	37	44.5 (MDR sample)		
T	14.1	4–8 (ca 6)	14	13.7	36.6	23.9
Ural	12.8	6.5	5.6	13.6	6.8	2.3
LAM	10.3	25	25	8	8.9	3.8
LAM-RUS	9.0	24	25			
Haarlem	5.1	4–8 (ca 6)	11	7.3	29.2	7.6
Other	2.6	5	0	7.4	17	47.2

^a^Karelia: Single isolates (X and unknown) are not shown

Settings (references): Pskov [18]; St. Petersburg [11, 20, 25]; Murmansk [24], Finland [17]

Beijing and LAM families are found prevalent in all Karelian regions (except for Pudozh and Ladoga but this data may be biased by small sample sizes). Beijing genotype is prevalent among *M. tuberculosis* strains circulating in Karelia but its prevalence in Finland remains low in spite of close trans-border contacts between Finland and St. Petersburg and Karelia. Furthermore, Beijing genotype isolates in Finland are likely not to be associated with Russian contact but rather with an increasing immigration from Asia [17, 28]. We observe similar and low prevalence rates of LAM family in Finland and adjacent Russian Karelia and Murmansk that is lower than in St. Petersburg and Pskov.

While Beijing and LAM present two the most prevalent families of *M. tuberculosis* across Russia, the Ural family was suggested as both endemic and low-prevalent lineage in Northern Eurasia (~ Former Soviet Union) (13). As far as this study is concerned, the rate of the Ural family appears to decrease in Finland vs Karelia. Furthermore, within Karelia the West–east increasing gradient of the Ural genotype is observed with the highest rate in the southeast (Fig. 1). Notably, half of the Ural family strains were isolated in a single south-easternmost Pudozh district. It should be noted that even in the early 1920s (when proportion of Karels in Russian Autonomous Republic of Karelia was relatively high, 40 %); the population of this district was almost 100 % Russian [29]. In this regard, the Ural family may justly be regarded as a “Russian” strain brought to Karelia through relatively long-term human influx from Russia.

In contrast to the Ural family, SIT40, the largest non-Beijing type, appears to be a historical Karelian strain which distribution is marked with decreasing rate both to the West (Finland and Sweden) and East/North (Archangel and Murmansk) of Karelia. SIT40 is rare elsewhere on a global scale (*n* = 112, according to SITVIT_WEB), it is found on different continents, but mainly in Europe (Italy, Austria, Germany, Denmark, Netherlands, USA, at 0.2–0.7 %). Among Northern neighbors of Karelia it is found in a very low percent in Finland, Sweden and in the northern Russian regions of Murmansk and Archangel to the North and East of Karelia (Table 5). Interestingly, we found one SIT40 isolate (STR-INH-resistant) in the 2006, mainly drug-resistant collection in Karelia, Olonets region (Pryazha district) in a newly diagnosed elderly patient born in 1942. This is the same area, namely, southern Karelia, where most of the SIT40 isolates were detected by the current study. Noteworthy, Karels constitute 37 % of population in the Pryazha district (compared to 7.5 % in all Russian Republic of Karelia) and the rate of rural, less mobile population is as high as 74 % [30]. In view of the above, we speculate that SIT40 may present a historical clone in the Karelia area in northern Europe. Based on the whole genome (new generation) sequencing of one SIT40 strain, it was assigned to the Euro-American lineage and SNP-barcode 4.1 according to Coll et al. [31] (Igor Mokrousov, Ekaterina Chernyaeva, Anna Vyazovaya, unpublished observations).

Finally, we feel that a minor note should be added about one particular spoligotype identified in one LAM strain in the new collection. It is SIT266 highly prevalent in Belarus [10, 32, 33] that is not a neighbor of Karelia. Interestingly, the proportion of Belarusians increased in Karelia from 0.9 % in 1939 to 10.9 % in 1959. Although many Belarusians returned to their home country in the 1960s [14, 34], it could be that an isolate of the “Belarusian” genotype SIT266 may represent a heritage of that past event 50 years ago.

### Drug resistance

Our results on drug resistance rates are in line with earlier studies carried out in different parts of the former Soviet Union that have demonstrated an association of the Beijing genotype with MDR and a continuously increasing rate of MDR among Beijing strains. In northwestern Russia, the MDR rate among Beijing strains from new TB cases was 79.8 % in Murmansk [24], 64.5 % in Kaliningrad [35], 79.3 % in Pskov [18]. In other parts of Russia it was somewhat lower, 46.9 % in Ural [36] and 34 % in Siberia [37]. With further evolution of the TB epidemic in Russia, the MDR phenotype is evolving to XDR, and consequently, this association is repeated for XDR [38, 39]. One can hardly expect an improvement of the situation in the country in view of the (i) unchanged *M. tuberculosis* population structure with increasing prevalence of the drug resistant variants and (ii) unchanged (insufficient) TB control measures.

A closer look inside the Beijing family revealed a special role of the cluster named B0/W148 and termed a “successful Russian clone” [11]. The increasing active circulation of this Beijing variant appears to be one of the major causes behind increasing rate of MDR-TB in Karelia in the last 8 years. Indeed a meta-analysis of different studies across the Former Soviet Union demonstrated highest propensity of B0/W148 to acquire drug resistance even when compared to the other Beijing variants [11]. A recent spinal TB study also highlighted a crucial capacity of the Beijing B0/W148 strains to disseminate to other sites [40].

On the other hand, drug susceptible group was and remains to be dominated by non-Beijing genotypes. In this regard, an intriguing decrease of the prevalence of the LAM family within the drug resistant group from 18.0 % (11/61) in 2006 to 8.7 % (4/46) in 2014 (*P* = 0.2) may be noted. This result looks unusual since the LAM family was demonstrated to be associated with drug resistance in other parts of Russia [18, 22].

Finally, the Ural family has been considered a low-virulent genotype of *M. tuberculosis* (reviewed in Mokrousov [41]) and is at low rate in Karelia. However even for the Ural genotype, similarly to the Beijing genotype, we observe both quantitative and qualitative worrisome changes: 1 polyresistant isolate (out of 61 drug resistant) in 2006 versus 3 MDR isolates (out of 46) in 2014. Again, this observation is in line with recent alarming trends in evolution of this “less exciting” family of *M. tuberculosis*. While meta-analysis showed a lack of association with drug resistance of these strains in the former USSR [13], very recent reports from different countries in Eastern Europe with apparently different National TB control programs describe an emergence of the MDR Ural strains [40, 42].

## Conclusions

Despite decreasing incidence of TB in Karelia in the last years, the situation with drug resistant TB continues to worsen. The most hazardous strains and clones (Beijing B0/W148 cluster being the major threat) furthermore dominated by MDR and pre-XDR isolates, increasingly spread within the population. Contrasting phylogeographic patterns have been revealed for the Ural genotype and SIT40 spoligotype; they may reflect a complex demographic history of Karelia within the course of the 20^th^ century.

## References

[1] Markelov YM (2011). Clinical and epidemiological features multidrug-resistant and the reasons for its spread in the Republic of Karelia.

[2] Markelov Y, Narvskaya O (2010). Circulation of multidrug-resistant tuberculosis pathogen strains in the Republic of Karelia. Probl. Tuberk. Bolezn. Legk..

[3] The Official Karelia. http://www.gov.karelia.ru/News/2012/05/0528_12_e.html. Accessed 19 November 2015.

[4] Eskelinen H, Alanen A (2012). Migration from Russia to Eastern Finland. Trudy Karelskogo nauchnogo Centra RAS.

[5] WHO 2012. Updated critical concentrations for first-line and second-line DST (as of May 2012) WHO-Stop TB Programme, Policy guidance on drug-susceptibility testing (DST) of second-line antituberculosis drugs WHO/HTM/TB/2008.392. World Health Organisation, Geneva. 26290924

[6] van Embden JDA, Cave MD, Crawford JT, Dale JW, Eisenach KD, Gicquel B (1993). Strain identification of Mycobacterium tuberculosis by DNA fingerprinting: recommendations for a standardized methodology. J. Clin. Microbiol..

[7] Mokrousov I, Rastogi N (2015). Spacer-based macroarrays for CRISPR genotyping. Methods Mol Biol..

[8] Gibson AL, Huard RC, Gey van Pittius NC, Lazzarini LC, Driscoll J, Kurepina N (2008). Application of sensitive and specific molecular methods to uncover global dissemination of the major RDRio sublineage of the Latin American-Mediterranean Mycobacterium tuberculosis spoligotype family. J Clin Microbiol..

[9] Dubiley S, Kirillov E, Ignatova A, Stepanshina V, Shemyakin I (2007). Molecular characteristics of the Mycobacterium tuberculosis LAM-RUS family prevalent in Central Russia. J Clin Microbiol..

[10] Mokrousov I, Vyazovaya A, Narvskaya O (2014). Mycobacterium tuberculosis Latin American-Mediterranean family and its sublineages in the light of robust evolutionary markers. J Bacteriol..

[11] Mokrousov I (2013). Insights into the origin, emergence, and current spread of a successful Russian clone of Mycobacterium tuberculosis. Clin Microbiol Rev..

[12] Ogarkov OB, Medvedeva TV, Zozio T, Pogorelov V, Nekipelov OM, Gutnikova MY (2007). Molecular typing of Mycobacterium tuberculosis strains of Irkutsk area (Eastern Siberia) in 2000–2005. Mol Meditsina (Moscow).

[13] Mokrousov I (2015). Mycobacterium tuberculosis phylogeography in the context of human migration and pathogen’s pathobiology: Insights from Beijing and Ural families. Tuberculosis (Edinb)..

[14] Anonymous. 2009. On migration of population in Karelia in the 20th century. http://knk.karelia.ru/2009/02/migracii.html Accessed 19 November 2015. In Russian

[15] Evacuation of Finnish Karelia. https://en.wikipedia.org/wiki/Evacuation_of_Finnish_Karelia. Accessed 19 November 2015.

[16] Sužiedelis SA (1981). On OSS report on wartime population changes in the Baltic. Lituanus.

[17] Smit PW, Haanperä M, Rantala P, Couvin D, Lyytikäinen O, Rastogi N (2013). Molecular epidemiology of tuberculosis in Finland, 2008–2011. PLoS One.

[18] Mokrousov I, Vyazovaya A, Otten T, Zhuravlev V, Pavlova E, Tarashkevich L (2012). Mycobacterium tuberculosis population in northwestern Russia: an update from Russian-EU/Latvian border region. PLoS One..

[19] Afanas’ev MV, Ikryannikova LN, Il’ina EN, Kuz’min AV, Larionova EE, Smirnova TG (2011). Molecular typing of Mycobacterium tuberculosis circulated in Moscow, Russian Federation. Eur J Clin Microbiol Infect Dis..

[20] Chernyaeva E, Dobrynin P, Pestova N, Matveeva N, Zhemkov V, Kozlov A (2012). Molecular genetic analysis of Mycobacterium tuberculosis strains spread in different patient groups in St. Petersburg, Russia. Eur J Clin Microbiol Infect Dis.

[21] Groenheit R, Ghebremichael S, Pennhag A, Jonsson J, Hoffner S, Couvin D (2012). Mycobacterium tuberculosis Strains Potentially Involved in the TB Epidemic in Sweden a Century Ago. PLoS ONE.

[22] Ignatova A, Dubiley S, Stepanshina V, Shemyakin I (2006). Predominance of multi-drug-resistant LAM and Beijing family strains among Mycobacterium tuberculosis isolates recovered from prison inmates in Tula Region, Russia. J Med Microbiol..

[23] Krüüner A, Hoffner SE, Sillastu H, Danilovits M, Levina K, Svenson SB (2001). Spread of drug-resistant pulmonary tuberculosis in Estonia. J Clin Microbiol..

[24] Mäkinen J, Marjamäki M, Haanperä-Heikkinen M, Marttila H, Endourova LB, Presnova SE (2011). Extremely high prevalence of multidrug resistant tuberculosis in Murmansk, Russia: a population-based study. Eur J Clin Microbiol Infect Dis..

[25] Narvskaya O, Mokrousov I, Otten T, Vishnevsky B, Read MM (2005). Molecular markers: application for studies of Mycobacterium tuberculosis population in Russia. Trends in DNA fingerprinting research.

[26] Toungoussova OS, Mariandyshev A, Bjune G, Sandven P, Caugant DA (2003). Molecular epidemiology and drug resistance of Mycobacterium tuberculosis isolates in the Archangel prison in Russia: predominance of the W-Beijing clone family. Clin Infect Dis..

[27] Tracevska T, Jansone I, Broka L, Marga O, Baumanis V (2002). Mutations in the rpoB and katG genes leading to drug resistance in Mycobacterium tuberculosis in Latvia. J Clin Microbiol..

[28] The Finnish Immigration Service. http://www.migri.fi/about_us/statistics/statistics_on_citizenship. Accessed 19 November 2015.

[29] Puudožin kihlakunta [Pudozh district]. https://fi.wikipedia.org/wiki/Puudo%C5%BEin_kihlakunta. Accessed 19 November 2015. In Finnish.

[30] Prääsän kansallinen piiri [Pryazha national municipality]. https://fi.wikipedia.org/wiki/Pr%C3%A4%C3%A4s%C3%A4n_kansallinen_piiri. Accessed 19 November 2015. In Finnish.

[31] Coll F, McNerney R, Guerra-Assunção JA, Glynn JR, Perdigão J, Viveiros M (2014). A robust SNP barcode for typing *Mycobacterium tuberculosis* complex strains. Nat Commun.

[32] Vasilenko N, Vyazovaya A, Mokrousov I, Limeschenko E, Semenov V, Narvskaya O (2006). Spacer oligonucleotide typing of drug-resistant Mycobacterium tuberculosis circulating on the territory of Belarus. Immunopat Allergol Infektol.

[33] Zalutskaya A, Wijkander M, Jureen P, Skrahina A, Hoffner S (2013). Multidrug-resistant Mycobacterium tuberculosis caused by the Beijing genotype and a specific T1 genotype clone (SIT No. 266) is widely transmitted in Minsk. Int J Mycobacteriol.

[34] Republic of Karelia. https://en.wikipedia.org/wiki/Republic_of_Karelia. Accessed 19 November 2015.

[35] Mokrousov I, Otten T, Zozio T, Turkin E, Nazemtseva V, Sheremet A (2009). At Baltic crossroads: a molecular snapshot of Mycobacterium tuberculosis population diversity in Kaliningrad, Russia. FEMS Immunol Med Microbiol..

[36] Umpeleva TV, Kravchenko MA, Eremeeva NI, Vyazovaya AA, Narvskaya OV (2013). Molecular genetic characterization of strains of Mycobacterium tuberculosis, circulating in the Ural region of Russia. Infekcia Immunitet..

[37] Dymova MA, Kinsht VN, Cherednichenko AG, Khrapov EA, Svistelnik AV, Filipenko ML (2011). Highest prevalence of the Mycobacterium tuberculosis Beijing genotype isolates in patients newly diagnosed with tuberculosis in the Novosibirsk oblast, Russian Federation. J Med Microbiol..

[38] Dymova MA, Cherednichenko AG, Alkhovik OI, Khrapov EA, Petrenko TI, Filipenko ML (2014). Characterization of extensively drug-resistant Mycobacterium tuberculosis isolates circulating in Siberia. BMC Infect Dis..

[39] Vyazovaya A, Mokrousov I, Zhuravlev V, Solovieva N, Otten T, Vishnevsky B (2015). Dominance of the Beijing genotype among XDR Mycobacterium tuberculosis strains in Russia. Int J Mycobacteriol..

[40] Vyazovaya A, Mokrousov I, Solovieva N, Mushkin A, Manicheva O, Vishnevsky B (2015). Tuberculous spondylitis in Russia and prominent role of multidrug-resistant clone Mycobacterium tuberculosis Beijing B0/W148. Antimicrob Agents Chemother..

[41] Mokrousov I (2012). The quiet and controversial: Ural family of Mycobacterium tuberculosis. Infect Genet Evol..

[42] Crudu V, Romancenco E, Noroc E, Alexandru S, Niemann S, Lange C (2014). Beijing and H4/Ural genotypes of M. tuberculosis are predominating among M&XDRTB patients in Moldova. Int J Tuberc Lung Dis.

